# Effects of Concentration and Temperature of Nutrient Solution on Growth and Camptothecin Accumulation of *Ophiorrhiza pumila*

**DOI:** 10.3390/plants9060793

**Published:** 2020-06-25

**Authors:** Ji-Yoon Lee, Miki Hiyama, Shoko Hikosaka, Eiji Goto

**Affiliations:** 1 Graduate School of Horticulture, Chiba University, Matsudo, Chiba 271-8510, Japan; jiyoon@chiba-u.jp (J.-Y.L.); miki-hiyama@chiba-u.jp (M.H.); goto@faculty.chiba-u.jp (E.G.); 2 Plant Molecular Research Center, Chiba University, Chiba, Chiba 260-0856, Japan

**Keywords:** hydroponics, medicinal plant, monoterpenoid indole alkaloid, plant factory with artificial lighting, root-zone environments

## Abstract

The medicinal plant, *Ophiorrhiza pumila*, naturally grows on the floors of humid inland forests in subtropical areas. It accumulates camptothecin (CPT), which is used as an anti-tumor agent, in all organs. We investigated the optimal hydroponic root-zone environments for growth and CPT accumulation in *O. pumila* in a plant factory. In experiment 1, to determine the appropriate nutrient solution concentration (NSC), *O. pumila* was cultivated using four concentrations (0.125, 0.25, 0.5, and 1.0 times) of a commercial solution for 63 days after the start of treatment (DAT). The electrical conductivity of these NSCs was 0.6, 0.9, 1.5, and 2.7 dS m^−1^, respectively. The total dry weights at 0.25 and 0.5 NSCs were higher than those at the other two NSCs. CPT content at 0.25 NSC was significantly higher than those at other NSCs. In experiment 2, to investigate an appropriate nutrient solution temperature (NST), *O. pumila* was cultivated at four NSTs (10, 20, 26, and 35 °C, named as T10, T20, T26, and T36, respectively) for 35 DAT. The growth and CPT content at T20 was the highest among the treatments. Therefore, root-zone environments of 0.25 NSC and 20 °C of NST produced the best growth and CPT accumulation in *O. pumila*.

## 1. Introduction

In developed countries, the number of cancer patients and the use of anticancer drugs are increasing with an increase in the aged population. Camptothecin (CPT) is one of the raw materials used in critical anticancer drugs. CPT induces cell death by inhibiting DNA topoisomerase Ⅰ [1,2]. CPT belongs to the monoterpenoid indole alkaloid group and was initially identified in extracts from *Camptotheca acuminata* (Nyssaceae) [1,3]. Recently, semi-synthetic derivatives of CPT have been widely used worldwide as clinical anti-tumor agents against cancers of the lungs, cervix, ovaries, colon, etc. [4,5,6]. The world market for these CPT derivatives reached 2.2 billion US dollars in 2008 and is expected to continue to increase in the future [7,8]. 

Despite its high demand in anticancer drugs, CPT has been extracted mainly from two arboreous plants, *C. acuminata* and *Nothapodytes foetida* (Olacaceae), that have a slow growth rate and low plant productivity [7,8,9]. As arboreous plants require a large cultivation area and high photosynthetic photon flux density (PPFD), open-field cultivation is necessary. However, it is difficult to produce and supply stable raw materials for drugs from open-field cultivation due to the unpredictability of factors such as seasonal change, variable weather, and pests [7,9]. Therefore, to meet the increasing demand for CPT, an alternative cultivation method needs to be developed or an alternative medicinal plant species must be found. 

*Ophiorrhiza pumila* (Rubiaceae) is a herbaceous perennial plant that produces CPT and its related alkaloids in all its organs and is viewed as a valuable alternative to arboreous sources [7,9]. It grows in mountainous, moist, shady habitats in subtropical forests in Ryukyus in southern Japan, Taiwan, southern China, northern Vietnam, and the Philippines [10]. *O. pumila* is a compact plant 5–20 cm high with a low PPFD requirement. This means that it can be cultivated in smaller spaces such as greenhouses or plant factories with artificial light (PFAL). 

To obtain a stable supply of plants and raw materials for drugs, a PFAL is an ideal cultivation system compared to open-field, or even a greenhouse. Plants of uniform quality can be cultivated throughout the year in a PFAL because environmental conditions, such as light, temperature, and humidity, can be controlled, regardless of the outside climate, while in a greenhouse the incident solar light cannot be controlled [11]. A PFAL with multi-layer cultivation shelves is suitable for plants less than 30 cm in height, such as leafy vegetables and seedlings, and provides high plant productivity per unit area [11,12]. Therefore, a PFAL may well be a suitable cultivation system for CPT production from *O. pumila*. However, there is little information on the cultivation environments needed for *O. pumila* in a PFAL. 

Hydroponic systems that provide sufficient water and nutrition to plants are essential tools for stable cultivation in a PFAL. Optimization of the nutrient solution concentration (NSC) for plant growth is the first step in establishing cultivation methods. NSC significantly affects the absorption of water and nutrients by the roots. In particular, excessively low or high NSC often causes a nutritional deficit or unbalanced absorption of mineral nutrients by roots, respectively. Many researchers have studied the optimal NSCs for the growth of horticultural crops [13,14] and medicinal plants [15]. As wild *O. pumila* is grown under nutrient-poor conditions, it is hypothesized that the optimal electrical conductivity (EC) of the nutrient solution for growth will be lower than that of horticultural crops. An important second step is establishing the optimal NSC for CPT accumulation of *O. pumila*.

The root-zone temperature is also known to be an effective factor for plant growth. For instance, when the nutrient solution temperature (NST) increases, the respiration rate of the roots increases, which often inhibits plant growth due to respiration exhaustion. Low NST also inhibits plant growth because it suppresses water absorption by roots. Moreover, NSTs of 5–15 °C were found to induce oxidative stress through the suppression of water absorption and increase the ascorbic acid concentration and sugar content in spinach [16] and the bioactive compounds in red perilla [17] and canola [18]. Previous studies reported that oxidative stress imposed by, for example, treatment with hydrogen peroxide (H_2_O_2_), can induce the accumulation of indole alkaloids by root cultures and cell suspensions of the medicinal plant *Uncaria tomentosa* [19,20]. Therefore, it is thought that NSTs that induce oxidative stress may increase the CPT content of *O. pumila*. In addition, because the main organs of CPT accumulation in *O. pumila* are the stems and roots [21], it is possible that the NST, which influences the root-zone, will affect the CPT accumulation in *O. pumila*. 

Therefore, the objectives of this study were to investigate the effects of the NSC (experiment 1; Exp. 1) and the NST (experiment 2; Exp. 2) on the growth and CPT accumulation of *O. pumila* in a PFAL.

## 2. Results

### 2.1. Experiment 1—Nutrient Solution Concentration (NSC)

#### 2.1.1. Electronic Conductivity, Plant Growth Characteristics, and Chlorophyll Concentration

**Figure 1:**
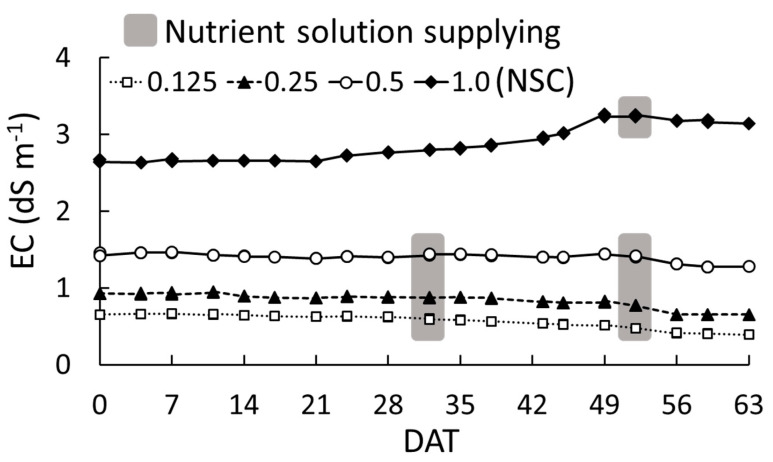
Change in electrical conductivity (EC) in each nutrient solution concentration (NSC) for 63 days after the start of the treatment (DAT) in experiment 1. The treatments were 0.125, 0.25, 0.5, and 1.0 NSC. The 1.0 NSC was the standard concentration of Otsuka-A nutrient solution. The grey marked DATs indicate the timing of the supply of the nutrient solution.

The EC at all NSCs was maintained at approximately the set value throughout the experiment (Figure 1). The EC at 1.0 NSC (the standard concentration of Otsuka-A nutrient solution) increased slightly from 21 days after the start of the treatment (DAT) and increased more evidently from 45 to 49 DAT, reaching 3.2 dS m^−1^. At 0.125, 0.25, and 0.5 NSCs, the EC was stable and decreased slightly from 35 DAT. From 0 to 56 DAT, the absorbed amount of the nutrient solution at 1.0 NSC was 1 L (daily absorbed rate was 1 mL per plant) and those at the other three treatments were 2.0 L (daily absorbed rate was 2 mL per plant).

**Figure 2:**
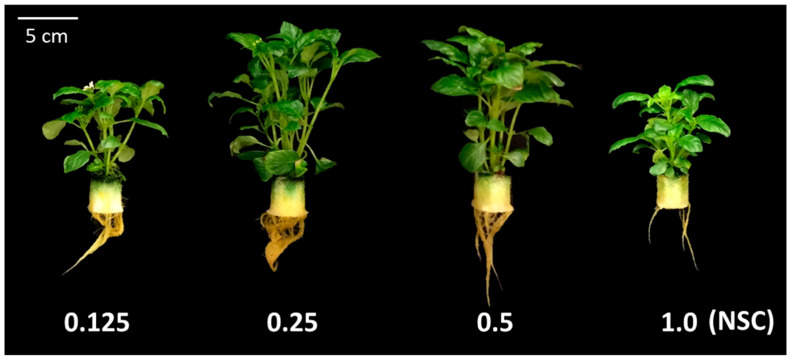
Pictures of *O. pumila* grown in each nutrient solution concentration (NSC) at 63 days after the start of the treatment (DAT) in experiment 1. The treatments were 0.125, 0.25, 0.5, and 1.0 NSC. The 1.0 NSC was the standard concentration of Otsuka-A nutrient solution.

At 63 DAT, the fresh weights of young leaves and stems at 0.25 and 0.5 NSCs were significantly higher than those at 0.125 and 1.0 NSCs (Table 1 and Figure 2). As the fresh weight of stems occupied the largest proportion among the organs in all treatments, the total fresh and dry weights at 0.25 and 0.5 NSCs were also significantly higher than those at the other two NSCs. There was no significant difference in the fresh weights of mature leaves and roots among the treatments.

**Figure 3:**
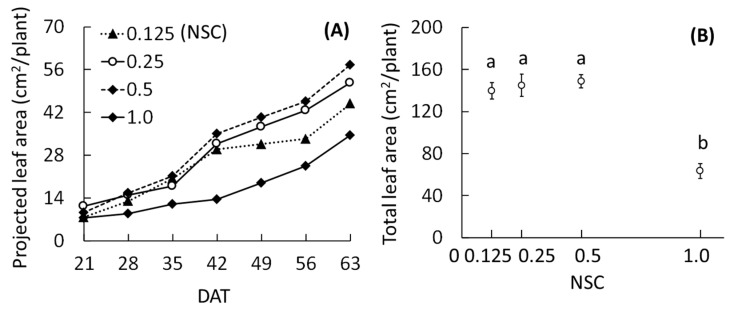
Effects of nutrient solution concentration (NSC) on projected leaf area (**A**) and total leaf area (**B**) of *O. pumila* in experiment 1. The projected leaf area was measured every week after 21 days after the start of the treatment (DAT), and the total leaf area was measured at 63 DAT. The treatments were 0.125, 0.25, 0.5, and 1.0 NSC. Vertical bars indicate S.E. (*n* = 8). Different letters indicate significant differences among the treatments at *p* < 0.05 by Tukey–Kramer’s test.

The projected leaf areas in all treatments except for 1.0 NSC increased rapidly from 35 to 42 DAT, after which those at 0.25 and 0.5 NSCs increased more rapidly than the other two treatments (Figure 3A). The rate of increase of the projected leaf area at 0.125 NSC was lower from 42 DAT onwards as compared to those at 0.25 and 0.5 NSCs. The projected leaf area at 1.0 NSC was the lowest among the treatments throughout the experiment. The rate of increase of the projected leaf area at 1.0 NSC was especially low until 42 DAT; thereafter, the rate was similar to 0.25 and 0.5 NSCs. At 63 DAT, the total leaf areas at 0.125, 0.25, and 0.5 NSCs were higher than that at 1.0 NSC (Figure 3B).

**Figure 4:**
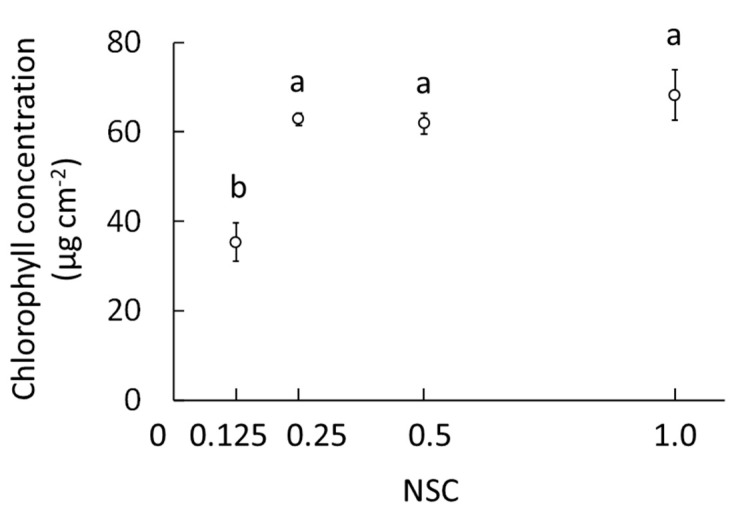
Effects of nutrient solution concentration (NSC) on the chlorophyll concentration in young leaves of *O. pumila* at 63 days after the start of the treatment (DAT) in experiment 1. The treatments were 0.125, 0.25, 0.5, and 1.0 NSCs. Vertical bars indicate S.E. (*n* = 8). Different letters indicate significant differences among the treatments at *p* < 0.05 by Tukey–Kramer’s test.

The chlorophyll concentration at 0.125 NSC was significantly lower than that at the other NSCs, approximately half of that observed with the other three treatments (Figure 4). The chlorophyll concentration was saturated at approximately 65–70 μg cm^−2^ at treatments above 0.25 NSC.

#### 2.1.2. Camptothecin

In all treatments, the highest CPT concentration was found in the stems, followed by that in the roots, young leaves, and mature leaves (Table 2). In young leaves, the CPT concentrations at 0.25 and 0.5 NSCs were significantly higher than those at 0.125 and 1.0 NSCs. Moreover, in the roots, the CPT concentration at 0.25 NSC was 1.6 times higher than those at the other NSCs. In mature leaves and stems, there was no significant difference in CPT concentration among the treatments.

**Figure 5:**
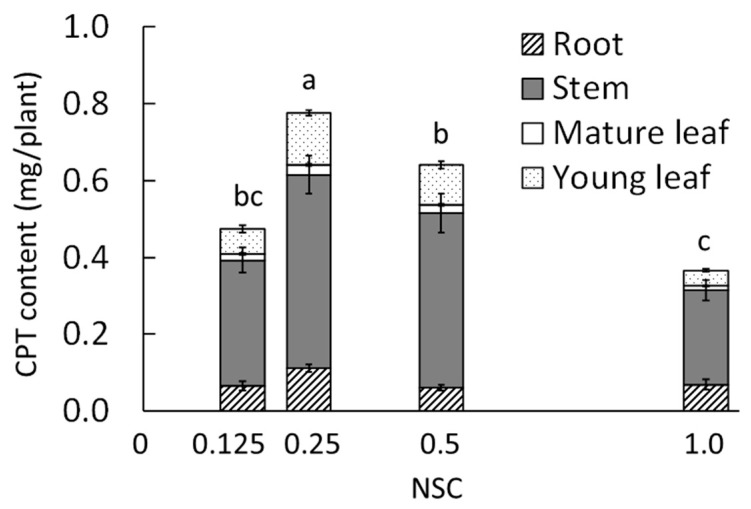
Effects of nutrient solution concentration (NSC) on the camptothecin (CPT) content in the whole *O. pumila* plant at 63 days after the start of the treatment in experiment 1. The treatments were 0.125, 0.25, 0.5, and 1.0 NSC. The CPT content was calculated using the CPT concentration determined by HPLC and the dry weight of each organ. Vertical bars indicate S.E. (*n* = 8). Different letters indicate significant differences among the treatments at *p* < 0.05 by Tukey–Kramer’s test.

The CPT content was significantly higher at 0.25 NSC (Figure 5). In all treatments, the stems had the highest CPT content among all the organs. 

### 2.2. Experiment 2—Nutrient Solution Temperature (NST)

#### 2.2.1. Plant Growth Characteristics

At T36, 50% of the treated plants died by 14 DAT, and 80% of them died by 35 DAT (data not shown). Furthermore, by 35 DAT, the plants showed marked damage, and the root color had changed to brown compared to the other treatments. Therefore, the results of T36 were omitted from this study. 

On 35 DAT, total fresh and dry weights and leaf area were significantly higher at T20 than with the other treatments. The fresh weight of the others, comprising the reproductive organs that include flowers, ovaries, and seed pods, was significant (Table 3). As the NST decreased, the dry matter ratio of the shoots and roots tended to increase, and thus, the dry matter of the roots at T10 was significantly higher than that at T26. 

**Figure 6:**
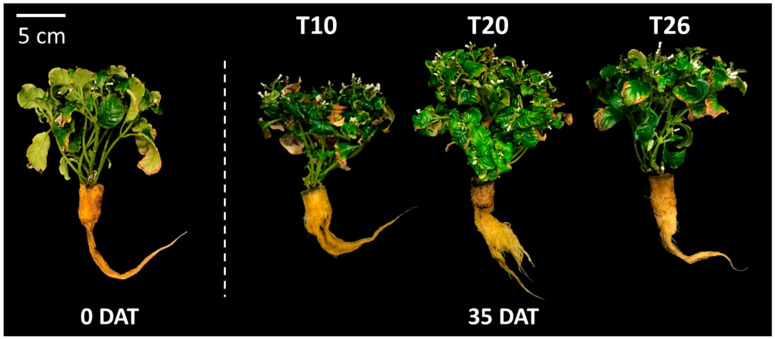
Pictures of *O. pumila* treated at each nutrient solution temperature (NST) at 0 and 35 days after the start of the treatment (DAT) in experiment 2. The NST treatments were set at 10, 20, and 26 °C as T10, T20, and T26, respectively. The NSTs at T10 and T20 were maintained using a handy cooler, and that at T26 was not controlled.

The plants at T20 showed vigorous growth and bore many lateral shoots and flowers (Figure 6). On the other hand, most of the mature leaves at T10 seemed to be aged and brown in color by 35 DAT unlike with the other treatments.

#### 2.2.2. Camptothecin

The CPT concentration in the roots was the highest among the organs, regardless of the NST (Table 4). The CPT concentrations in the stems and roots were two to three times higher than those in the leaves regardless of the NST. The CPT concentration in the young leaves was not affected by NST treatment, but the concentration in the mature leaves was significantly higher at T10 than for the other NSTs. In the stems and roots, the CPT concentrations at T10 and T20 were significantly higher than those at T26.

**Figure 7:**
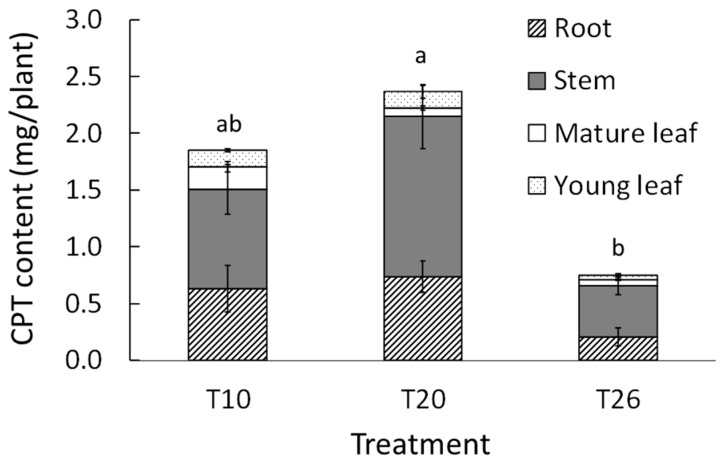
Effects of nutrient solution temperature (NST) on the camptothecin (CPT) content in the whole *O. pumila* plant at 35 days after the start of the treatment (DAT) in experiment 2. The NST treatments were set at 10, 20, and 26 °C as T10, T20, and T26, respectively. The NSTs at T10 and T20 were maintained using a handy cooler, and that at T26 was not controlled. The CPT content was calculated using the CPT concentration determined by HPLC and the dry weight of each organ. Vertical bars indicate S.E. (*n* = 3). Different letters indicate significant differences among the treatments at *p* < 0.05 by Tukey–Kramer’s test.

The parts that contributed most to the CPT content were the stems and roots in all treatments (Figure 7). The total CPT contents at T10 and T20 were higher than that at T26. The CTP content at T20 was approximately three times higher than that at T26 and this was statistically significant. 

## 3. Discussion

### 3.1. Experiment 1—Nutrient Solution Concentration (NSC)

In this study, we found that the 0.25 and 0.5 NSCs were more suitable for growing *O. pumila* than the 0.125 and 1.0 NSCs. It is known that excessively high or low concentrations of a nutrient solution negatively affects plant growth, yield, and quality [14,15]. Kang and van Iersel [22] found that the growth and flower quality of salvia (*Salvia splendens*) showed a peak at 1.0 times of Hoagland solution (EC 2.0 dS m^−1^), while at 0.125, 0.25, and 2.0 times (EC 0.4, 0.7, and 3.7 dS m^−1^), they were lower. According to our results, the change in the EC, projected leaf area, and absorbed amount of the nutrient solution explained the differences in water uptake rate and growth of *O. pumila* among the treatments. At 1.0 NSC, the EC was stable or slightly increased, while the decrease in the volume of the nutrient solution and the increase rate of projected leaf area was low compared to those of the other treatments until 42 DAT. This suggested that the plant at 1.0 NSC absorbed little of the nutrient solution until 42 DAT because of osmotic stress and that the EC in 1.0 NSC was higher than the absorption concentration of *O. pumila*. Other researchers have also reported that high NSC and high EC caused a reduction in plant growth through osmotic stress [14,23,24]. However, after 42 DAT, the growth inhibition at 1.0 NSC was alleviated and the rate of increase of the projected leaf area at 1.0 NSC was the same as those of the other treatments. Therefore, it is possible that the roots of *O. pumila* acclimated to a high NSC in the latter growth stage and that the optimal NSC may change depending on the nutritional conditions in the place of origin, plant varieties, and growth stage. 

At 42 DAT, the 0.125 NSC in this study showed a deficit in the nutrient elements required by *O. pumila*. As, in our experiment, the volume of the nutrient solution in each treatment was the same, the total amount of mineral nutrition was the lowest at 0.125 NSC. This NSC might have an insufficient supply of minerals such as N and Mg for growth and chlorophyll production in *O. pumila* compared to the 0.25 and 0.5 NSCs. Ding et al. [23] reported that the chlorophyll content of pakchoi (*Brassica campestris* L. ssp. Chinensis) significantly decreased at lower concentrations of nutrient solution (EC 0–0.6 dS m^−1^). Our results indicated that both the maximum growth and a high chlorophyll concentration (indicating a high photosynthetic ability) were obtained when *O. pumila* was grown in 0.25 and 0.5 NSCs (EC 0.9 and 1.5 dS m^−1^, respectively). 

In our study, the CPT concentrations of the young leaves and roots were higher at 0.25 and 0.5 NSCs, where growth was promoted, than at 0.125 and 1.0 NSCs. It is known that secondary metabolites are synthesized from primary metabolites; therefore, the NSC for growth promotion may also be appropriate for CPT accumulation in *O. pumila*. Although there was no difference in growth between the 0.25 and 0.5 NSCs, the CPT concentration in the roots at 0.25 NSC was significantly higher than that at 0.5 NSC. Roots are one of the main organs for CPT production in *O. pumila* [21]. The 0.25 NSC treatment might be suitable for not only the nutrient and water uptake but also for CPT biosynthesis or accumulation in the roots of *O. pumila*. Further experiments are needed to elucidate the mechanism of CPT accumulation by NSC. 

The CPT content at 0.25 NSC was significantly higher than those at the other NSCs because the total dry weight and CPT concentration of young leaves and roots were the highest at this NSC. Within the range of NSCs and the treatment period in this experiment, 0.25 NSC was the most suitable NSC for plant growth and CPT accumulation in *O. pumila*. 

### 3.2. Experiment 2—Nutrient Solution Temperature (NST)

It is known that a root-zone temperature that is too high for optimal growth will decrease the photosynthetic rate [25], increase the respiration rate, and change the absorption of water and nutrients by the roots [26]. In creeping bentgrass, when the roots were exposed to 35 °C, the respiration rate of the whole plant exceeded the net photosynthetic rate of the canopy, resulting in growth inhibition [27]. In our study, we found that NSTs of 26 and 36 °C significantly inhibited growth compared to 20 °C NST, although the habitat of wild *O. pumila* is subtropical [10]. An NST of 36 °C was too high to maintain the growth of *O. pumila* even if the NST treatment period was 14 days. It is possible that the main reason for growth inhibition at T36 is an excessive respiration rate in roots because the root respiration rate at 36 °C may be almost 2.5 times as high as that at 20 °C, according to the theory of temperature coefficients (Q10) [28]. 

At low NSTs, Sakamoto et al. [29] reported that a 10 °C and a 20 °C root-zone temperature increased both total biomass and reproductive organs (including the fruits) of strawberries, compared to 30 °C. For temperate crops, many researchers report photosynthesis inhibition, leaf area reduction, and growth inhibition caused by reducing the water absorption by roots exposed to 12 °C in the case of cucumber [30], and 14 °C in the case of rice [31]. From our results, an NST of 20 °C seemed to be more suitable for vegetative growth and reproductive growth in *O. pumila*, compared to 10 °C. The plant growth and the fresh weight of the others were significantly higher at T20 than those at T10 and T26. In addition, in *O. pumila*, most of the mature leaves at T10 withered, and the dry matter ratios of the shoots and roots were the highest out of all the NSTs. According to these results, the water absorption of *O. pumila* may have been suppressed at 10 °C NST, which inhibited growth even though there was sufficient water in the root-zone. 

In our unpublished data, the air temperature of 28 °C used in this experiment was the optimal condition for the photosynthesis, transpiration, and the growth of above-ground parts of *O. pumila* [32]. Therefore, these findings showed that the combination of different temperature controls for the root-zone (20 °C) and above-ground parts (28 °C) was more suitable to maximize the whole plant growth in *O. pumila*.

In this study, the CPT concentrations of all organs at T26 were markedly lower than those at T10 and T20. It is suggested that the high respiration rate of the roots at T26 negatively affected not only plant growth but also CPT accumulation. Srivastava et al. [33] mentioned that the availability of precursors through primary photosynthetic metabolites markedly influenced alkaloid accumulation. This suggests that CPT as an alkaloid accumulation at T26 may also require primary photosynthetic metabolites.

In general, low NST inhibited water absorption and often induced water stress, thus generating ROS. This ROS generation is known to be a trigger of secondary metabolite production in many vegetables [16] and medicinal plants [17,18]. Although there are few reports on alkaloid production under low NST, Malik et al. [34] reported that the expression levels of alkaloid biosynthetic enzymes and target alkaloid accumulation in all organs of *Catharanthus roseus* and the roots of *Nicotiana tabacum* were higher at root-zone temperatures of 12 °C than at 25 and 30 °C. In our experiment, at T10, the plants showed a typical water stress phenomenon because of low NST; however, the CPT concentration at T10 was the same as that at T20. These results indicate that 10 °C NST did not promote CPT accumulation in any of the organs of *O. pumila* compared to 20 °C. 

It should be noted that the NST treatment in Exp. 2 started 120 days after transplantation to a hydroponic container, i.e., when the plants were bigger and more mature than in the NSC treatment in Exp. 1. The difference in CPT concentrations of 0.25 NSC and T26, which are the same NSC and NST conditions, is thought to be related to the growth stage (Table 2 and Table 4).

To produce the raw materials for drugs, it is important to understand and increase the total amount of target compounds as well as overall growth. In all the treatments in our experiment, we found that the stems and roots were the main organs contributing to a high CPT content compared to the other organs. The CPT content at T20 was higher than that at T10, although CPT concentrations at T10 and T20 were the same. Thus, it is thought that an NST of 20 °C is optimal for both growth and CPT accumulation in *O. pumila* because of the adequate absorption rate and the respiration rate of the roots.

In conclusion, at the optimal air temperature of 28 °C for the above-ground parts, the optimal NSC and NST were 0.25 and 20 °C, respectively, to improve the growth and CPT accumulation of *O. pumila*. These findings highlight the necessity for investigations to determine an appropriate NSC level to maximize CPT yields from *O. pumila*. Moreover, different temperature controls for the root-zone and above-ground parts can further improve plant growth and CPT accumulation. Our research suggests that *O. pumila* is a valuable plant for the production of CPT as an antitumor agent, and that PFAL is a suitable cultivation system for CPT production from *O. pumila*. 

## 4. Materials and Methods 

### 4.1. Plant Material and Growth Conditions

The original *O. pumila* plants were propagated by tissue culture. The upper portion of plants, which had two leaves, were cut off and the cuttings were transplanted onto a urethane sponge. To acclimate the cuttings and wait for root emergence, plants were watered using tap water, and the relative humidity (RH) was kept at approximately 100%. 

Three weeks after cutting, the seedlings with four leaves and 50–100 mg of total fresh weight were transplanted to a container with a nutrient solution and the NSC treatment mentioned below was started. *O. pumila* was cultivated under a transparent plastic cover to maintain the RH at 90%. The environmental conditions except for the NSC and NST were kept as follows: 28 °C air temperature, 16-h light period, PPFD of 100 ± 5 μmol m^−2^ s^−1^ with white light emitting diode lamps, and CO_2_ concentration of 1000 μmol mol^−1^. PPFD was measured near the growth point of the plants.

### 4.2. Treatment Plots

#### 4.2.1. Experiment 1—Nutrient Solution Concentration (NSC)

The nutrient solution used for this experiment was a commercial product, Otsuka-A nutrient solution (OAT Agrio Co., Ltd., Tokyo, Japan), which is widely used for leafy and fruit vegetable cultivation in Japan. The composition for a standard concentration (one-strength) was 16 mM NO_3_^−^, 4 mM H_2_PO_4_^3−^, 4 mM Ca^2+^, 2 mM Mg^2+^, 8 mM K^+^, and 1.3 mM NH_4_^+^ and a micronutrient solution. The EC of the standard concentration of Otsuka-A was 2.7 dS m^−1^ (including the EC of tap water) and is called 1.0 NSC in this study. The pH of this composition was 5.5–6.5. Since *O. pumila* is a wild plant with a slow growth rate compared to horticultural crops, the NSC treatments were set at lower concentrations than that of the 1.0 NSC. Four treatments were set in total at 0.125, 0.25, 0.5, and 1.0 NSCs and the experiment continued for 63 days. The ECs of these treatments were 0.6, 0.9, 1.5, and 2.7 dS m^−1^, respectively. 

Twenty seedlings for each treatment were transplanted to a container filled with 4 L of the nutrient solution. When the volume of nutrient solution in the container decreased to approximately 80% (3 L), further nutrient solution at the original concentration was supplied to bring each container back up to 4 L. The additional nutrient solution in all treatments except for 1.0 NSC was done at 32 and 56 DAT, and that of 1.0 NSC was done at 56 DAT. The pH of all nutrient solutions was adjusted to maintain 6.5 using a portable pH meter (Cyberscan pH 310, Eutech Instruments, Singapore), every 3–4 days. The EC of each nutrient solution was measured using a portable conductivity meter (Cyberscan CON 400, Eutech Instruments, Singapore) at the same time as the pH adjustment. In this experiment, NST was approximately 26 °C without any control under the air temperature of 28 °C. To supply sufficient air to roots and to circulate the nutrient solution in each container, aeration was performed using air pumps and air stones.

#### 4.2.2. Experiment 2—Nutrient Solution Temperature (NST)

The seedlings were cultivated hydroponically for 120 days under the same environmental conditions as Exp. 1. Based on the results of Exp. 1, the NSC in the containers was 0.25 NSC of Otsuka-A. Ten plants with an average 14.2 g of total fresh weight were transplanted to a container filled with 10 L solution of 0.25 NSC, and the NST treatments mentioned below were started at 0 DAT (Figure 6).

The NST treatments were set at 10, 20, 26, and 36 °C (as T10, T20, T26, and T36, respectively), which were lower and higher temperatures around the ambient NST (26 °C). The NSTs, except for T26, were maintained and controlled using a handy cooler (TRL-107NHF, Tomas Kagaku Co., Ltd., Tokyo, Japan) and a thermal controller (TC-107, Tomas Kagaku Co., Ltd., Tokyo, Japan). Additionally, to keep the NST uniform and to supply air to the roots, aeration was performed using air pumps and air stones. The NST in the containers was measured by two thermocouples in each treatment and recorded by a data logger at 15-min intervals. 

### 4.3. Plant Growth Characteristics 

All of the growth parameters mentioned below were measured at 63 DAT in Exp. 1 and at 0 and 35 DAT in Exp. 2. In both experiments, each organ of *O. pumila* plants with 4–6 stems, in which each stem had 4–6 nodes, was classified as follows to measure the fresh and dry weights and analyze CPT (Figure 2 and Figure 6). The leaves from the top to the second nodes were classified as young leaves, and the leaves below that were classified as mature leaves. In Exp. 1, all stems and flower buds were classified as a stem, because the flower buds were too small to be analyzed for CPT. On 0 and 35 DAT, in Exp. 2, all reproductive organs, including flowers, ovaries, and seed pods, were classified as “Others”, because the mature plants bore many flowers and seeds. As the numbers of flowers and ovaries varied among the plants in each treatment, the fresh and dry weight of others were measured, but their CPT was not analyzed. 

In Exp. 1, for all treatments, the different organs from sampled plants were stored in a freezer at −30 °C to analyze the concentration of chlorophyll and CPT. At the same time, the different organs from other plants were dried at 50 °C for 72 h in a convection oven (MOV-112F drying chamber, Panasonic Cor., Osaka, Japan) to measure the dry weight. In Exp. 2, each organ was lyophilized for 24 h using a freeze-dryer (FDU-1110, Tokyo Rikakikai, Tokyo, Japan). After measurement of the dry weight of a lyophilized organ, CPT was analyzed using the same organ because the number of plants was limited. 

Total leaf area was calculated from a photograph of all separated leaves from the stem using free imaging software (LIA 32 ver. 0.378). The projected leaf area in Exp. 1 was calculated every week using the same software from a photograph taken from the top of the plant canopy. 

### 4.4. Chlorophyll Concentration

In Exp. 1, the chlorophyll concentration was determined by the method of Porra et al. [35]. The young leaves were disrupted using a Multi-beads shocker (MB-601U, Yasui Kikai Corporation, Osaka, Japan). The powdered sample (approximately 20 mg) was weighed and incubated at room temperature in 1 mL of N, N-dimethylformamide for two days to extract the chlorophyll. The chlorophyll concentration was determined by measuring the absorbance of leaf extracts at 663.8, 646.8, and 750 nm with a spectrophotometer (V-550, JASCO Corporation, Tokyo, Japan), and was expressed per unit leaf area by using the specific leaf area in each treatment.

### 4.5. Camptothecin Analysis

CPT concentration was analyzed according to the method of Asano et al. [9] with some modifications for our study. Through a preliminary experiment, the ratio of extraction solution volume to sample was optimized depending on the water content in the sample, i.e., frozen (fresh) in Exp. 1 and lyophilized (dried) in Exp. 2.

The powdered sample of each organ (100 mg of fresh weight and 50 mg of dry weight) was mixed with 1 mL of methanol (≥99.9%) in a 2 mL tube and extracted with an ultrasonic washer (ASU-2, As One Corporation, Japan; output of 40 W) for 15 min. The extracted sample was stored overnight at 4 °C. On the next day, the extracted sample was separated using a centrifuge (MX-305, Tomy Seiko Co., Ltd., Tokyo, Japan) at 10,000 *g* for 10 min. The supernatant was filtered through a syringe filter (13HP020AN, Advantec, Japan) and analyzed by HPLC (10AD, Shimadzu Corporation, Kyoto, Japan).

A TSK gel ODS-100V column (Tosoh, Japan; 4.6 × 250 mm, 5 mm) was used with a solvent system of methanol:water (7:3, v/v). The flow rate of the mobile phase in the column was 1.0 mL min^−1^ for 25 min, and the injection volume was 10 μL per sample. The column temperature was set at 40 °C. The chromatogram was monitored at 254 nm on an ultraviolet-visible photodiode array detector (SPD-M10A, Shimadzu Corporation, Kyoto, Japan). Camptothecin (Sigma-Aldrich, St. Louis, MO, USA) was used as a standard material.

### 4.6. Statistical Analysis

Data were statistically evaluated by one-way analysis of variance (ANOVA) with the SPSS program for Windows (Version 24.0; SPSS Inc., Chicago, US). To investigate significant differences among treatments, the means of measurement parameters were compared using Tukey–Kramer’s test at *p* < 0.05.

## Figures and Tables

**Table 1:** Effects of nutrient solution concentration (NSC) on fresh and dry weights of *Ophiorrhiza pumila* at 63 days after start of the treatment (DAT) in experiment 1 (*n* = 8).

NSC	Fresh Weight (g)					Total Dry Weight (mg)
	Leaf ^1^		Stem	Root	Total	
	Young	Mature				
0.125	0.9 b ^2^	1.0	1.7 b	0.7	4.3 b	36.6 b
0.25	1.3 a	1.1	2.9 a	0.7	6.0 a	51.3 a
0.5	1.3 a	1.1	2.9 a	0.8	6.0 a	46.3 a
1.0 ^3^	0.5 b	0.8	1.2 b	0.7	3.2 b	31.5 b

^1^ Leaves from the top to the second node were classified as young leaves, and the leaves below that were classified as mature leaves. ^2^ Different letters within columns indicate significant differences among the treatments at *p* < 0.05 by Tukey–Kramer’s test. ^3^ 1.0 NSC is the standard concentration of Otsuka-A nutrient solution.

**Table 2:** Effects of nutrient solution concentration (NSC) on camptothecin concentration of *O. pumila* organs at 63 days after the start of the treatment (DAT) in experiment 1 (*n* = 8).

NSC	Camptothecin Concentration (mg g^−1^ DW)			
	Leaf ^1^		Stem	Root
	Young	Mature		
0.125	0.8 b ^2^	0.1	2.2	1.0 b
0.25	1.2 a	0.2	2.0	1.7 a
0.5	1.0 a	0.2	2.0	0.9 b
1.0 ^3^	0.7 b	0.1	1.9	1.0 b

^1^ Leaves from the top to the second node were classified as young leaves, and the leaves below that were classified as mature leaves. ^2^ Different letters within columns indicate significant differences among the treatments at *p* < 0.05 by Tukey–Kramer’s test. ^3^ 1.0 NSC is the standard concentration of Otsuka-A nutrient solution.

**Table 3:** Effects of nutrient solution temperature (NST) on growth of *O. pumila* at 35 days after start of the treatment (DAT) in experiment 2.

DAT	NST (°C)	Fresh Weight (g)						Total Dry Weight (g)	Dry Matter Ratio (%)		Leaf Area (cm^2^)
		Leaf ^1^		Stem	Others ^3^	Root	Total		Shoot	Root	
		Young	Mature								
0		0.5	2.6	3.8	2.3	2.5	14.2	1.4	10.8	4.3	35.9
35	T10	1.3	3.0	4.5	4.5 b ^2^	4.7	18.0 b	2.9 b	19.4	7.2 a	151.7
	T20	1.4	5.3	7.4	9.7 a	6.2	30.1 a	4.6 a	17.7	6.0 ab	254.2
	T26 ^4^	1.1	4.1	6.3	3.3 b	4.2	18.9 b	2.6 ab	16.3	5.2 b	202.0

^1^ Leaves from the top to the second node were classified as young leaves, and the leaves under that were classified as mature leaves. ^2^ Different letters within columns indicate significant differences among the treatments on 35 DAT at *p* < 0.05 by Tukey–Kramer’s test (*n* = 3). ^3^ Others are reproductive organs that included flowers, ovaries, and seed pods. ^4^ NST at T26 was not controlled.

**Table 4:** Effects of nutrient solution temperature (NST) on camptothecin concentrations in *O. pumila* at 35 days after the start of the treatment (DAT) in experiment 2.

NST (°C)	Camptothecin Concentration (mg g^−1^ DW)			
	Leaf ^1^		Stem	Root
	Young	Mature		
T10	0.5	0.3 a ^2^	1.3 a	1.8 a
T20	0.5	0.1 b	1.4 a	2.0 a
T26 ^3^	0.4	0.1 b	0.8 b	1.0 b

^1^ Leaves from the top to the second node were classified as young leaves, and the leaves below that were classified as mature leaves. ^2^ Different letters within columns indicate significant differences among the treatments on 35 DAT at *p* < 0.05 by Tukey–Kramer’s test (*n* = 3). ^3^ NST at T26 was not controlled.
